# The Application of Hemospray in Gastrointestinal Bleeding during Emergency Endoscopy

**DOI:** 10.1155/2017/3083481

**Published:** 2017-01-23

**Authors:** Alexander F. Hagel, Heinz Albrecht, Andreas Nägel, Francesco Vitali, Marcel Vetter, Christine Dauth, Markus F. Neurath, Martin Raithel

**Affiliations:** ^1^Department of Gastroenterology, University of Erlangen, Ulmenweg 18, 91054 Erlangen, Germany; ^2^Institute for Employment Research, Regensburger Straße 104, 90478 Nuremberg, Germany; ^3^Department of Gastroenterology, Waldkrankenhaus St. Marien, Rathsberger Str. 57, 91054 Erlangen, Germany

## Abstract

*Introduction*. Gastrointestinal bleeding represents the main indication for emergency endoscopy (EE). Lately, several hemostatic powders have been released to facilitate EE.* Methods*. We evaluated all EE in which Hemospray was used as primary or salvage therapy, with regard to short- and long-term hemostasis and complications.* Results*. We conducted 677 EE in 474 patients (488 examinations in 344 patients were upper GI endoscopies). Hemospray was applied during 35 examinations in 27 patients (19 males), 33 during upper and 2 during lower endoscopy. It was used after previous treatment in 21 examinations (60%) and in 14 (40%) as salvage therapy. Short-term success was reached in 34 of 35 applications (97.1%), while long-term success occurred in 23 applications (65.7%). Similar long-term results were found after primary application (64,3%) or salvage therapy (66,7%). Rebleeding was found in malignant and extended ulcers. One major adverse event (2.8%) occurred with gastric perforation after Hemospray application.* Discussion*. Hemospray achieved short-term hemostasis in virtually all cases. The long-term effect is mainly determined by the type of bleeding source, but not whether it was applied as first line or salvage therapy. But, even in the failures, patients had benefit from hemodynamic stabilization and consecutive interventions in optimized conditions.

## 1. Introduction

Gastrointestinal bleeding (GIB) represents a potentially life-threatening condition and is a main indication for gastrointestinal endoscopy. Its incidence is given with roughly 150 patients per 100000 population and year, with a mortality rate of still 10% [1, 2]. Various bleeding sources may be identified and they may be induced either because of an underlying morbidity with ulcerative and nonulcerative lesions, respectively (e.g., ulcers, esophageal varices, and gastrointestinal vascular malformations), and malignancy or as a consequence of iatrogenic interventions (e.g., postpolypectomy bleeding) [3]. In addition, GIB becomes an emerging issue in the light of increasing numbers of disease entities requiring strict anticoagulant therapy with increasing use of new direct oral anticoagulants (DOAC) [4].

Hemostasis can be achieved according to the type of lesion and extent of bleeding by injection of saline, diluted epinephrine solution, macrogollaurylether (e.g., aethoxysklerol), the application of various types of through-the-scope (TTS) or over-the-scope clips (OTSC), or using argon-plasma-coagulation and other thermic coagulation procedures [5]. Despite these possibilities, 10–30% of all patients remain in which an endoscopic hemostasis cannot be obtained, or in which a prompt recurrence of the bleeding occurs [2, 6–8].

Recently, sprayable powders for induction of immediate bleeding stop were introduced in gastrointestinal endoscopy. The latest innovative system introduced into clinical use is the EndoClot system (EndoClot Plus Inc., Santa Clara, CA, USA) which consists of starch, while the Hemospray (Cook Medical, Winston-Salem, NC, USA) consisting of an inorganic powder is the most widely used chemical in this regard [5]. It exerts multimodal mechanisms to achieve hemostasis, becoming cohesive and adhesive after coming in contact with moisture hence forming a stable mechanical barrier, sealing the bleeding site. Due to its composition, it is neither absorbed nor metabolized within the mucosa, hence minimizing the risk of systemic toxicity [6].

Previous studies described Hemospray as a feasible and possible new option to obtain rapid hemostasis during gastrointestinal endoscopy either as primary treatment option [9–14] or as a salvage indication, when refractory bleeding persists despite application of other conventional methods (e.g., injection therapy and clips) [11, 14]. In these studies with defined inclusion and exclusion criteria, a high initial success of up to 100% is reported. However, rebleeding rates, depending on the bleeding source of up to 38.9%, are noted in literature [5]. The aim of this paper is to report the indications, experience, results, and adverse events from the use of Hemospray between August 2013 and November 2014 from a high-volume endoscopy university center investigating unselected consecutive emergency patients.

## 2. Material and Methods

### 2.1. Patient Collective

In this study, we included all consecutive patients in whom the use of Hemospray was deemed necessary for achieving successful hemostasis. Between August 2013 and November 2014 all emergency endoscopies (EE) for the indication of GIB were analyzed. Endoscopies were defined as EE when patients presented with signs of acute bleeding (e.g., coffee-ground or fresh blood vomit, melena, and perianal bleeding) and/or anemia (hemoglobin < 10 g/dL) and/or compromised cardiovascular parameters (hypotonia, tachycardia). Emergency endoscopies fulfilling above listed criteria were routinely conducted within 6 hours after initial presentation.

Depending on the suspected bleeding source, either gastroscopy, colonoscopy, or both were performed within the emergency examination.

All patients resulted from daily routine or emergency schedule and represented nonselected patients arising from real-life conditions. In 27 patients, Hemospray was applied and they were thus enrolled for further detailed analysis.

Hemospray is not the first possible intervention during EE in our department when coagulation parameters are normal or moderately altered. In patients without severely disturbed coagulation, Hemospray was generally used as salvage therapy. In patients under therapeutic anticoagulation or with severely disturbed coagulation, Hemospray was also used as a primary therapeutic modality.

The data obtained was prospectively entered in a database and retrospectively evaluated. Approval for this study was obtained from the local ethics committee.

### 2.2. Indications and Performance of Emergency Endoscopy (EE)

EE was performed in standard manner with Olympus gastroduodenoscopes (GIF 1T160, GIFH180) and Pentax colonoscopes (FC-38M) in the case of acute GIB (hematemesis, hematochezia, and melena) and/or suspected GIB because of anemia and hypovolemic shock or deterioration of physical status in patients with known underlying GIB source. EE was performed during pethidine and midazolam or pethidine and propofol analgosedation, respectively. Erythromycin before EE was given to some patients after decision of the endoscopists on duty. Standard examination of gastroscopy or colonoscopy was performed with cleansing of each segment to identify the bleeding source(s).

Usually, conventional bleeding treatment was applied with a stepwise treatment approach, for example, (i) first injection techniques (epinephrine 1 : 100000, fibrin glue (Baxter, Unterschleißheim, Germany)); or (ii) in case of visible vessels, perforating or gapping lesions through-the-scope (TTS) or over-the-scope clips (OTSC) were tried (Olympus, Hamburg, or Ovesco, Tübingen, Germany); (iii) in variceal bleeding band ligation technique (6 Shooter Saeed Multi-Band-Ligator, Cook Medical, Bloomington, IN, USA) or injection of Histoacryl (n-butyl-2-cyanoacrylat/lipiodol mixture), respectively, was used; (iv) diffuse tumor bleeding or angiectasias were treated by argon-plasma-coagulation (BOWA Electronics Arc 400, Gomaringen, Germany, or ERBE VIO 200, Tübingen, Germany).

Following this stepwise endoscopic treatment approach using these modalities (i–iv), Hemospray was not intentionally used as the primary treatment option in most patients. Only in patients with diffuse mucosal bleeding combined with a severe coagulopathy (e.g., due to liver cirrhosis) or with therapeutic anticoagulation (e.g., intake of vitamin K antagonists (VKA) or direct oral anticoagulants (DOAC)) Hemospray was held as a possible option for primary bleeding treatment to avoid any risky tissue manipulation (table indications).

### 2.3. Hemospray Application

Hemospray was applied using the standard 10 French catheter which is supplied by the manufacturer (Cook Medical, Bloomington, Indiana, USA) [9]. Normally, Hemospray is applied in bursts, which release 1–5 g of powder each. In order to prevent sticking of the catheter tip in moisture and hence clotting of the catheter, release of powder was normally started with the tip being 2-3 cm away from the bleeding source.

### 2.4. Study Parameters, Definitions, and Statistics

The primary aim of this study was to assess whether Hemospray application resulted in successful bleeding treatment (short-term and long-term hemostasis) with stop of bleeding from the causal lesion at least for 5 minutes under direct visual endoscopic observation. These findings were each documented in the Viewpoint files and the clinical data register.

Secondary aims were recurrence rate, side effects, and outcome parameters in the patients treated with Hemospray.

Short-term success of bleeding treatment was defined as direct hemostasis, achieved during EE and no further bleeding signs within the next 24 hours.

Long-term success of bleeding treatment was defined as no further bleeding event and/or treatment for at least 30 days after the index EE.

However, another bleeding source than the originally treated one which may have occurred after or independently from the index EE did not affect registration of the long-term success related to the treated lesion.

## 3. Results

### 3.1. Frequency of Hemospray Requirement in Emergency Endoscopy (EE)

During the study period of 15 months 488 upper GI EE in 345 patients were registered. Twenty-seven of 345 patients with emergency GIB (7,8%) received at least one Hemospray application. In total, in these 25 patients 33 applications of Hemospray were recorded (1.2 Hemospray applications per patient).

In 344 of these 488 upper GI endoscopies, a bleeding source could be identified. In 158 examinations, ulcers have been found as bleeding source (Forrest Ia: 31, Ib 61, IIa 27, IIb 39). Variceal ligature was performed in 23 patients.

We conducted further 189 emergency colonoscopies in 130 patients. During two of these examinations (1.05%), Hemospray was applied.

In three patients, previous EE were conducted, with Hemospray being applied in a follow-up endoscopy. In one patient, an endoscopic intervention was conducted previously (ligature of esophageal varices with consecutive bleeding of the ligature ulcer). In two further examinations, diffuse gastral erosions in one patient and an erythematous anastomosis following gastrectomy were recorded, without initial bleeding signs. In follow-up endoscopies, diffuse bleeding instances in these areas were found and a primary Hemospray application was conducted.

### 3.2. Bleeding Sources, Patient Characteristics, Hemostasis, and Recurrence Rate

The characteristics of all patients in whom Hemospray was applied are listed in Table 1 according to their main indications and bleeding sources. Next to a plethora of GIB sources during upper GI endoscopy, two anastomotic ulcers in the colon were treated with a Hemospray application, which was performed as an off-label use. The different bleeding sources have been subdivided according to the following therapy in Table 2.

Hemostasis rates are listed in Table 3 and assigned to upper or lower GIB. Short-term success with hemostasis (24 h) was high with 97.1% and was recorded in nearly all applications except for one fatal bleeding case. However, long-term success decreased to 65.7% after 30 days of observation.

Recurrent bleeding was found after treatment with Hemospray after 11 examinations (31.4%) in 10 patients (37.0%).

### 3.3. Use of Hemospray as Salvage Treatment

Previous bleeding treatment was performed in 21 of 35 examinations (60.0%). Here, Hemospray was used as salvage therapy because of failure of conventional bleeding treatment. Thus, among all EE performed during this time period 4,3% (21/488 EE) presented as real refractory emergency bleeding.

Previous bleeding treatment was conducted in bleeding esophageal varices by ligature but did not achieve the defined bleeding stop; thus the ligatures were combined with Hemospray for definitive hemostasis. In bleeding ulcers, injection of epinephrine with/without fibrin glue with/without the application of hemoclips was performed without effect until finally Hemospray was used.

Previous endoscopic therapy can be split into two categories. In seven patients, one procedure was conducted (ligature of esophageal varices and injection of fibrin glue in one patient each and injection of diluted suprarenin solution in five patients). Main indications of a sole injection therapy were diffuse larger bleeding sources without visible vessels. Here, the hemostatic powder was estimated as superior to mechanical or thermic alternatives.

In eight patients, two interventions were futile in achieving hemostasis. In all patients, diluted suprarenin was used, in combinations with through-the-scope (TTS) clips (four patients), over-the-scope clips (OTSC) (one patient), and the additional injection of fibrin glue (in three patients).

**Figure 1:**
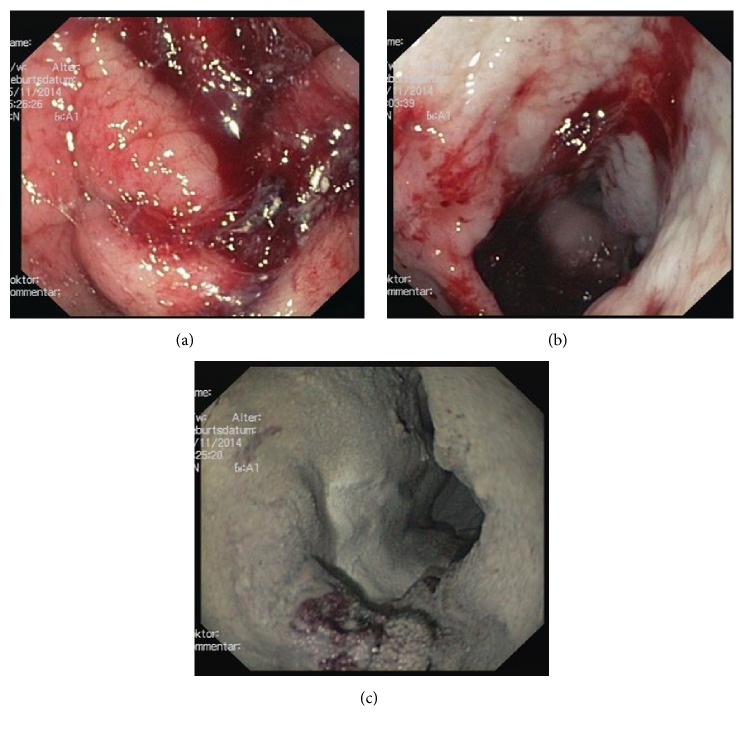
(a)–(c): acute antral bleeding (a), which cannot be terminated by injection of diluted epinephrine solution (200 mL in total) (b). After the application of Hemospray, hemostasis can finally be achieved (c).

In six patients, even a combination of diluted suprarenin solution and two further measures was insufficient in achieving hemostasis. In detail, the combination of TTS and argon-plasma-coagulation (APC) was used in two patients, the additional combination of fibrin glue and OTSC in one patient, and TTS and fibrin glue in three patients. The main problems of futile mechanical measures were mainly ulcers in the posterior wall of the duodenum, in which the bleeding area could not be grasped completely or oozing bleeding remained after previous therapy (Figures 1(a)–1(c)).

### 3.4. Hemospray as Primary Hemostasis Method

In the remaining 14 examinations (40%), Hemospray was used as singular approach (primary treatment) without previous conventional treatment steps because of (at least) one of the following reasons:

1 of 27 patients (3.7%) required Hemospray application because of analgosedation failure with an extremely restless patient. Hemospray was applied to at least terminate the bleeding temporarily in order to improve sedation and vital parameters.

One fulminant arterial bleeding instance (3,7%) from the gastroduodenal artery required primary Hemospray to terminate bleeding temporarily and to gain some time for preparation of an emergency surgical procedure in stable conditions.

Further primary Hemospray applications were done in 4 patients (14,8%) presenting with diffuse gastric or duodenal bleeding in which the bleeding source could not be detected.

Finally, severely impaired coagulation due to liver cirrhosis or therapeutic anticoagulation stipulated Hemospray in 6 patients (22,2%).

### 3.5. Hemospray Application in Patients with Impaired Coagulation

Fifteen examinations (42,9%) were conducted with an impaired coagulation system. An impaired coagulation system was defined as thrombocytes < 50000/uL or an INR > 1,8 in an acute bleeding situation or the intake of NOAKs. In six patients, coagulation was altered medicamentously by Warfarin in two and Rivaroxaban in two patients. The coagulation was optimized in the patients on Warfarin with prothrombin concentrates before the examination. In these patients, endoscopic standard procedures were conducted and failed to achieve hemostasis. Hence Hemospray achieved hemostasis as salvage method. Both patients with Rivaroxaban showed diffuse bleeding. Here, Hemospray was successfully used as primary method.

Eight patients suffered from an impaired coagulation due to liver cirrhosis and one due to a liver transplant rejection. All of them received prothrombin concentrates, fresh frozen plasm, or thrombocyte concentrates, depending on the actual parameters. In five, first line interventions failed, while Hemospray was used as first treatment in four patients.

### 3.6. Analysis of Clinical Conditions with and without Hemospray Efficiency

Overall, Hemospray proved to obtain at least a short-term hemostasis in virtually all cases (34/35, 97.1%). Only in one patient (3.7%), in whom an esophageal carcinoma caused an aortoesophageal fistula with massive torrential bleeding, EE was performed during cardiopulmonal resuscitation. Here the application of Hemospray was ineffective because of the massive extent of bleeding. Hemospray application during resuscitation measures was further technically ineffective because of lacking space with air in the tubular esophagus flooded with blood from the aorta, causing repeated clotting of the application catheter.

In 16 of 27 patients (59.3%) in which Hemospray could be applied, a permanent success could be achieved without any rebleeding within 30 days from the same bleeding source. In the remaining 10 of 27 patients (37.0%) recurrent bleeding was encountered in patients either with extremely deep ulceration which had eroded an artery (mainly the gastroduodenal artery) or with malignant lesions.

At least, in these patients, the application of Hemospray stabilized the patients' conditions; hence further treatment (clip application in a second endoscopy, radiologic coiling of the corresponding artery, or a surgical procedure) could be conducted in a stable setting. Two of these patients suffered from gastric carcinoma causing diffuse bleeding. In these patients, rebleeding occurred, albeit all endoscopic measures were exhausted. In one patient, a gastrectomy was performed; in the second, coiling of the gastroduodenal artery was performed, terminating the GIB permanently.

In two patients suffering from esophageal varices, Hemospray was applied. In both, ligature was conducted as first line therapy. While in one, oozing bleeding was seen directly after the intervention, the second patient showed initially a sufficient hemostasis with recurring bleeding signs (fresh blood in the gastral probe) after 6 hours. As correlate, oozing bleeding next to one of the ligatures could be identified. In both, Hemospray application terminated the bleeding immediately and permanently. Hence further rescue interventions, for example, TIPS, could be spared.

### 3.7. Analysis of Mortality Cases from Gastrointestinal Bleeding (GIB)

Of note, in this cohort with refractory or severe GIB 5 out of 27 patients (18.5%) died during the hospital stay. Three patients (11.1%) died due to septic multiorganic failure, not related to EE. In these patients, death was not related to endoscopy or the GIB in any means.

In one patient, suffering from ethyl-toxic cirrhosis (3.7%), a duodenal ulcer caused an erosion of the gastroduodenal artery. In this patient, numerous endoscopies were performed in order to achieve hemostasis. Hemospray was only effective for achieving short-term hemostasis but could not induce long-term efficacy. Since this was not successful, the ulcer was even treated surgically twice. But no intervention was successful to cease the intermittent GIB including surgical measures. The patient developed several further complications, for example, renal failure and pneumonia; thus the medical treatment was then limited in due course.

In the fifth patient, an esophageal carcinoma caused an esophago-aorto fistula, requiring massive blood transfusions and mechanical life support. During EE the Hemospray proved to be ineffective in view of the massive bleeding and life resuscitation and, technically, the catheter could not be deployed appropriately due to clotting of the Hemospray within the catheters. Hence, no hemostasis could be reached and life support was terminated due to the infaust prognosis in this patient.

### 3.8. Major Adverse Events from Hemospray Application

In one patient (3.7%), EE was conducted due to melena following ischaemic colitis with complete colectomy and consecutive generalized peritonitis. The gastric anterior wall presented additionally with diffuse bleeding. Due to the compromised anticoagulation, Hemospray was administered. Immediately after the application, a new recess with white and fatty tissue appeared and could be documented. Hence laparotomy was performed rapidly after EE. Here an 8 cm perforation in this area was found and sewed. This major adverse event was closely related to Hemospray application. It seems possible that the forces which emerge during the release of the powder might have ruptured the tissue and caused the perforation of an apparently inflamed tissue.

## 4. Discussion

Hemospray is a new device for the treatment of GIB. Its efficient use in achieving hemostasis has been described as first line therapy previously [15, 16]. In our endoscopy department, Hemospray is normally only used as first line therapy in patients with a severely disturbed coagulation system. Otherwise, it is applied as salvage therapy, when other routine measures for the treatment of acute GIB fail (e.g., injection of epinephrine or fibrin, mechanical therapy by application of TTS or OTS clips, and thermal therapy). In various studies, a combination of these interventions has shown better short- and long-term effects in achieving hemostasis during EE [17, 18]. Hence a combination of two modalities is recommended in endoscopic guidelines [19]. In our patients, in which Hemospray was used as salvage therapy, a combination of at least two of the above listed traditional tools has normally been applied without achieving hemostasis. An exception presented patients with diffuse mucosal bleeding. After futile injection therapy, Hemospray was applied in several patients instead of mechanical or thermal alternatives.

When analyzing the cases where Hemospray was used as salvage therapy after unsuccessful conventional treatments, a subpopulation of 4.3% of all patients was identified among all EE with severe refractory GIB. Hence we represent real-life data of an unselected patient collective, since bleeding in most EE can be terminated by one or a combination of the mentioned methods. In these 4.3% of patients we found a substantial number of severe underlying diseases, such as liver cirrhosis and advanced stages of malignancies, complicating endoscopic hemostasis. If Hemospray would not have been available during these interventions, these patients would have very likely required emergency surgical or urgent radiological treatment. Thus, Hemospray represents a valuable emergency tool for approximately 5% of all patients with progredient GIB.

In our cohort, in total, Hemospray was found to be an efficient tool to achieve hemostasis during EE with a high short-term success rate of 97% and a moderate long-term success of 65,7%. Similar promising results have been described in literature [5, 9–11]. However, after the successful initial endoscopy, high rebleeding rates can be found, as we encountered in our population recurrent bleeding in 31% after all examinations. Thus, despite its rapid procoagulative and covering effects, Hemospray did not appear to stimulate wound healing or tissue remodeling rapidly. Similar comparable rebleeding rates of 20–40% have been found in previous studies [9–14, 20]. In these studies, the rebleeding rate could be related to additional risk factors, such as antithrombotic therapy or malignancies [4, 12]. In our cohort, we can also confirm futile long-term hemostasis in advanced malignancies due to often accompanying necrotic tissue, exposed vessels, and increased contact-vulnerability of the tissue. Furthermore, we have encountered problems with, especially, long-term hemostasis, in ulcerations with an eroded artery in our collective.

For the long-term failure in arterial bleeding, covering the arterial vessel by Hemospray powder lasted very likely just not long enough to enable a sufficient fibrin clot formation or subsequent reendothelialization. Hence, when the Hemospray was washed off, rebleeding occurred. Similarly, in malignancies, progredient tissue destruction and necrosis contributed to several recurrences of GIB after Hemospray used in these areas. However, the application of Hemospray can be seen as partially successful in these patients, especially with regard to enabling the endoscopists to create an intermittent hemostasis for a restricted time in order to buy time to stabilize the cardiovascular situation of the patient, to administer blood transfusions and/or coagulation factors if necessary, and to organize further therapy (e.g., surgery). However, Hemospray does not represent a fire and forget device which guarantees a long-term hemostasis in all patients. Ten of 27 patients (37.0%) developed recurrent rebleeding within the observation period of 30 days.

During daily routine, we found the utilization of Hemospray beneficial in patients with markedly reduced anticoagulation (e.g., Warfarin or DOAC, or due to underlying diseases). In these situations, injection therapy or the application of clips can cause mucosal damage or may increase vessel lesions accidentally, resulting in further increasing the likelihood of severe or recurrent GIB. In several patients with reduced anticoagulation, especially with diffuse bleeding, Hemospray was used as first line therapy to create hemostasis and showed a high long-term success in all of these cases. Interestingly, in the case of DOAC bleeding the high rate of short-term success by Hemospray is of great value, because all DOAC have short half-lives of around 10–12 hours except in severe renal insufficiency or hepatic failure [4]. The intermittent hemostasis provided by Hemospray in nearly all cases helps to overcome their therapeutic anticoagulative effects. Thereafter, coagulation normalizes and the likelihood increases that vascular lesions may be closed by endogenous fibrin formation or after repeated endoscopic therapy. Thus, DOAC induced diffuse bleeding without significant obvious pathology represents a further valuable indication for Hemospray application.

As described above, previous studies applied Hemospray either as primary or as salvage therapy. In our cohort, the application was based on the choice of the endoscopists. Hence, we can compare the results of both indications in our cohort. On the one hand, the indications for primary (diffuse or malignant bleeding, especially with temporarily compromised coagulation parameters) and salvage therapy (bleeding ulcers, in which injection and mechanical therapy were insufficient, especially in hard to reach lesions) differed. On the other hand, we have found similar long-term results, with rebleeding in more than one third of the patients in every subgroup. In these patients, larger (arterial) vessels in inflamed ulcers or diffuse malignant lesions will not heal sufficiently for endoscopic hemostasis alone. Here, hemostasis can be reached for the moment, buying time to stabilize the patient and proceed to further treatments (surgical or radiological).

During the study period of 15 months, we encountered one possible complication caused by the application of Hemospray. In the above described setting, the pressure caused by the CO2 release is deemed to be responsible for a perforation of the inflamed gastric wall. After Hemospray application an 8 cm large gastric perforation with view to the peritoneum appeared requiring surgery directly after gastroscopy. As reported from the literature this is a feared side effect of Hemospray application [14, 21], but this was the single one major complication resulting from 35 Hemospray applications in 27 patients (3,7%) in our population.

In our patient collective, we found a fairly high 30-day all-cause mortality rate of 18,5% with 5 deaths among 27 patients. However, only one death was directly caused by an untreatable massive GIB from a malignant aortoesophageal fistula, a condition where Hemospray was also ineffective due to technical reasons, bleeding extent and resuscitation. Bleeding related mortality was documented only in this patient (1/27; 3.7%). Thus, it is lower than reported from others in EE [3]. The other remaining fatalities resulted due to septic multiorganic failure in three (11,1%) and due to decompensated liver cirrhosis in one patient (3,7%), further demonstrating the number of challenges which are routinely encountered in this patient collective.

## 5. Conclusion

Hemospray enlarges the armament of emergency endoscopists. It is a safe and easy to use device which can be used in upper and lower endoscopy both as first line treatment and as salvage therapy with short-term effectiveness of 97%. However, it should not be assigned as a magic bullet, since long-term success was found to be only 60% after 30 days. We have encountered several rebleeding instances within 12–24 hours in up to 37% of patients, especially in malignancy or deep ulcerations of larger eroded arteries. These tend to rebleed after the elimination of Hemospray from the bleeding source. However, even these patients showed a benefit from the treatment, since intermittent bleeding cessation allows the stabilization of hemodynamic parameters and patients' clinical condition and gives time to plan onwards a definite treatment (e.g., surgery) in a controlled setting.

## Figures and Tables

**Table 1:** Patient characteristics and frequency distribution of the treatment indications. In several patients suffering from upper GI bleeding, more than one endoscopy involving hemostatic measures and the application of Hemospray was necessary. Hence, we recorded more examinations involving interventional measures than patients. Merely second look endoscopies without further necessary interventions have not been calculated here.

Male/female	19/8	

Age	Median 72 years, range 40–88 years	

	Patients	Examinations

Upper GI bleeding	25	33

Lower GI bleeding	2	2

*Bleeding source*		
Ulcers	13	18
Tumor	2	4
Postinterventional	3	4
Diffuse bleeding	6	6
Reflux esophagitis	2	2
Others	1	1
Total examinations	27	35

**Table 2:** Bleeding sources as noted during emergency endoscopy, divided according to the kind of Hemospray application (primary treatment versus salvage therapy).

	Primary therapy	Salvage therapy
*Bleeding source*		
Ulcers	6	11
Tumor	2	2
Postinterventional	1	2
Diffuse bleeding	3	3
Variceal bleeding	1	1
Reflux esophagitis	0	2
Others	1	0
Total examinations	14	21

**Table 3:** Short-term success was defined as successful hemostasis during endoscopy persisting for at least 24 hours. Long-term success was defined as no further bleeding from the treated bleeding source within 30 days. One patient died during EE due to an aortoesophageal fistula. Three more patients died due to septic multiorganic failure during the hospital stay; one patient died due to liver failure following cirrhosis. These four fatalities were not linked to emergency endoscopy.

	Per examination	Per patient
*Overall success*		
Short-term	34/35 (97.1%)	**26/27 (96.3%)**
Long-term	23/35 (65.7%)	**17/27 (63.0%)**

*Success upper GIB*		
Examinations	33	**25**
Short-term success	32 (97,0%)	**24 (96%)**
Long-term success	21 (63,6%)	**15 (60%)**

*Long-term success*		
*Primary therapy*	9/14 (64,3%)	
*Salvage therapy*	14/21 (66,7%)	

*Success lower GIB*		
Endoscopy	2	2
Examinations	2 (100%)	2 (100%)
Short-term success	2 (100%)	2 (100%)
Long-term success	2 (100%)	2 (100%)

*Unsuccessful treatment = recurrent bleeding*	**11/33 (33.3%)**	**11/25 (44.0%)**
Ulcers	9	9
Carcinoma	2	2

*Fatalities*	**5/35 (14.2%)**	**5/27 (18.5%)**
Bleeding associated	1	1
Others	4	4

*Further interventions*	**10/35 (28,6%)**	**10/27 (37,0%)**
*Emergency *surgery	3	3
Reendoscopy	6	6
Radiologic coiling	1	1

Technical failure	1/35 (2.8%) Clotting of catheter	1/27 (3.7%)

## Appendix

### Role of Hemospray and Its Indications in Emergency Endoscopy (EE)

Short-term hemostasis provided by Hemospray may give time

- for cardiovascular stabilization,
- for substitution of blood products (erythrocyte concentrates) or coagulation factors,
- for spontaneous recovery of coagulation parameters in DOAC-treated patient,
- to organize further semielective radiological or surgical treatment,
- to support the endoscopy team by experienced supervisors,
- to achieve rapid bleeding stop in restless patients, analog-sedation induced complications, or patients with high ASA scores (3-4).

*Favourable Hemospray Indications*

- Diffuse bleeding (e.g., erosions, anticoagulated patients, or coagulopathy)
  - Superficial to deep ulcers without significant erosion of a large artery
  - Ulcerated lesions with venous bleeding
  - Quick hemostasis in restless patients (e.g., alcohol withdrawal and high comorbidity)
- Bleeding after endoscopic interventions (e.g., large polypectomy and papillectomy).

*Unfavourable Hemospray Indications*

- Strong arterial bleeding and erosion of a submucosal or deeper located artery (e.g., gastroduodenalis)
  - Aortoesophageal fistula with great leakage.

*Relative Contraindications*

- Ischaemic or severely inflamed gastrointestinal wall
  - Lesions with suspected covered perforation
  - Location near diverticula.

## Abbreviations

DOAC: Direct oral anticoagulants
EE: Emergency endoscopy
GIB: Gastrointestinal bleeding
VKA: Vitamin K antagonists.
